# Methylation Risk Scores in Psychiatric Disorders: Advancing Epigenetic Research in Mental Health

**DOI:** 10.31662/jmaj.2024-0329

**Published:** 2025-02-28

**Authors:** Kazutaka Ohi, Daisuke Fujikane, Kentaro Takai, Ayumi Kuramitsu, Yukimasa Muto, Shunsuke Sugiyama, Toshiki Shioiri

**Affiliations:** 1 Department of Psychiatry, Gifu University Graduate School of Medicine, Gifu, Japan

**Keywords:** methylation risk score, epigenetics, schizophrenia, bipolar disorder, anxiety disorder

## Abstract

DNA methylation is an epigenetic modification implicated in psychiatric disorders influenced by both genetic and environmental factors. Methylation risk scores (MRSs) have emerged as a tool for quantifying accumulated epigenetic modifications and assessing the predisposed risk for certain common disorders. This narrative review introduces the MRS application in psychiatric disorders, including schizophrenia (SCZ), bipolar disorder (BD), social anxiety disorder (SAD), and panic disorder (PD), while also discussing the current limitations and ethical considerations in psychiatric research. MRSs are calculated from epigenome-wide association studies (EWASs) for psychiatric disorders in various tissues from blood and brain and reflect methylation patterns associated with the psychiatric disorder risk. MRSs provide a perspective of how the cumulative methylation patterns at specific CpG sites may contribute to the onset of psychiatric disorders. In SCZ and BD, MRSs derived from both blood and brain tissues have shown distinct methylation profiles that differentiate these disorders, particularly in patients with a high genetic SCZ risk. MRSs are also used to assess the impact of environmental factors, such as early-life adversity and chronic stress, on psychiatric outcomes. In SAD and PD, where epigenetic studies are relatively limited, MRSs revealed both shared and distinct epigenetic features between anxiety disorders, with specific methylation changes associated with social avoidance in SAD patients. MRSs can serve as biomarkers, providing a valuable understanding of both genetic predispositions and environmental influences on gene regulation. However, the lack of large-scale EWAS datasets and standardized summary statistics remains as a limitation. To address this issue, this review provides a list of publicly available raw intensity data (IDAT) files from psychiatric epigenetic studies that can help facilitate future research by providing the raw data necessary for conducting independent EWASs and MRS calculations. As the field advances, careful consideration must be given to the ethical implications of MRS applications, particularly in clinical intervention and prevention. While MRSs hold promise for future personalized medicine applications, informing treatment decisions based on an individual’s methylation profile, caution is warranted regarding their predictive utility and effect size limitations. This review emphasizes the importance of MRSs in advancing psychiatric research, bridging the gap between genetic risk and environmental factors.

## Introduction

Deoxyribonucleic acid (DNA) methylation is an epigenetic modification involving the addition of a methyl group to the cytosine residue primarily within cytosine-phosphate-guanine (CpG) dinucleotides without altering the DNA sequence. Methylation in the gene promoter regions generally suppresses gene expression, thereby influencing various biological processes ^(1), (2), (3), (4)^. Methylation can both be reversible and irreversible ^(1), (5)^. Reversible methylation occurs in response to environmental factors, such as stress or diet ^(5)^, while certain methylation patterns can be stably maintained throughout an individual’s lifespan, potentially influencing biological functions over time ^(1)^. Additionally, although evidence from animal models suggests that some methylation signals may be inherited across generations ^(6)^, this stable epigenetic inheritance has not been definitively proven in humans. In humans, most epigenetic signals are reprogrammed during early embryonic development ^(7)^, making it challenging to determine whether methylation patterns are directly transmitted from parents to offspring.

In psychiatric disorders, DNA methylation is implicated in the regulation of genes associated with brain development and function, contributing to the pathogenesis of disorders, such as schizophrenia (SCZ), bipolar disorder (BD), and anxiety disorders. These epigenetic modifications can be influenced by environmental factors, including early-life adversity, chronic stress, or trauma, which may interact with inherited genetic risks. Furthermore, methylation patterns exhibit tissue specificity, with notable differences between the blood and brain tissues ^(8)^, although some common patterns exist between these tissues ^(9)^. While blood-derived methylation data can provide valuable perspectives on psychiatric disorder and potential biomarkers for these disorders ^(10), (11), (12), (13), (14), (15)^. Brain tissue, particularly in regions, such as the prefrontal cortex and the hippocampus, may exhibit distinct methylation profiles that are not only related to cognitive and emotional regulation but also to psychiatric disorders. Thus, both blood and brain tissues provide valuable information for understanding the pathogenesis underlying psychiatric disorders ^(3), (4)^.

In recent years, the methylation risk score (MRS) concept has emerged as a powerful tool across various research fields ^(16), (17), (18)^, except for psychiatric research. MRSs quantify the cumulative effects of multiple differentially methylated CpG sites associated with diseases, providing a more comprehensive assessment of the epigenetic risk. By integrating data from epigenome-wide association studies (EWASs) for different conditions or diseases, MRSs can help identify individuals at a higher risk based on their unique methylation profiles. This approach offers valuable perspectives on how environmental factors may influence disease onset and progression. In this review, we introduce our methodology and application of MRSs in psychiatric research, highlighting available resources and discussing their potential role in elucidating the complex relationships between genetics and the environment in the etiology of psychiatric disorders.

## Overview of the Polygenic Risk Scores in Psychiatric Research

In psychiatric genetics research, the usage of polygenic risk scores (PRSs) is a well-established method for assessing an individual’s risk for developing psychiatric disorders based on the accumulation of small effects across thousands of genetic loci ^(19), (20), (21), (22), (23), (24), (25), (26), (27), (28), (29)^. The method evaluates the contribution of multiple single-nucleotide polymorphisms (SNPs) across the entire genome, integrating their effects to calculate the overall genetic risk score. The PRS reflects the cumulative genetic predisposition of an individual for developing psychiatric disorders, such as SCZ, BD, and other psychiatric disorders.

The process of calculating PRSs typically begins with discovery genome-wide association studies (GWASs) for identifying relevant genetic variants. For psychiatric disorders, international consortia, such as the Psychiatric Genomics Consortium (PGC), UK Biobank (UKBB), ENIGMA, and COGENT, have conducted large-scale GWASs, providing summary statistics on millions of SNPs associated with psychiatric disorders or related intermediate phenotypes ^(30), (31)^. One million SNPs in DNA samples from the own target cohort of psychiatric patients and healthy controls can be genotyped using data from GWAS arrays. Once the relevant SNPs are identified from the discovery GWAS summary statistics, PRSs are calculated by applying various thresholds (e.g., *P_T_* < 0.001, *P_T_* < 0.01,* P_T_* < 0.05, *P_T_* < 0.1, *P_T_* < 0.5, and *P_T_* ≤ 1) to select SNPs that are nominally associated with the disorder in the discovery GWAS. The effect size (odds ratio or *beta* coefficient) of each SNP is extracted, and the PRS is calculated as the weighted sum of these SNPs, with each SNP weighted according to its effect size in the target individuals. This process results in a genetic risk score reflecting the genetic susceptibility to a given psychiatric disorder or intermediate phenotype.

PRSs have become an invaluable tool in psychiatric research and practice because they provide a quantitative measure of the genetic risk that can be applied across diverse populations and datasets. For instance, individuals with higher PRS values for SCZ have a significantly increased risk of developing the disorder compared to those with lower scores ^(20), (26)^. These scores have also been used to predict cognitive impairments, brain structures, and other intermediate phenotypes associated with psychiatric disorders ^(20), (21), (22), (23), (25), (27), (28), (29)^, making them a powerful tool for both research and potential clinical applications.

## Purpose of MRSs in Psychiatric Disorders

While PRSs capture the inherited genetic risk for psychiatric disorders, MRSs provide a complementary perspective by focusing on epigenetic modifications occurring due to environmental influences. As MRSs have emerged as a valuable tool in the genetic research field, we have been able to apply the methodology to the psychiatric research field, allowing us to quantify the cumulative DNA methylation patterns associated with psychiatric disorders ^(32), (33)^. These MRSs integrate methylation patterns reflecting regulatory mechanisms influenced by both genetic predispositions and environmental factors, such as early-life adversity, trauma, chronic stress, or lifestyle factors ^(34)^.

The unique strength of MRSs lies in their ability to capture the dynamic interaction between genetic and environmental influences, which is particularly important for psychiatric disorders. Unlike PRSs, which are static and reflect the inherited risk, MRSs can provide perspectives on how the environment modulates genetic risk over time, potentially altering the trajectory of mental health conditions. For instance, individuals with a genetic predisposition to SCZ or BD may have varying methylation profiles depending on their exposure to early-life stressors or other environmental risk factors.

By analyzing data from discovery EWASs conducted in various tissues, such as blood or brain tissue, MRSs provide perspectives on how methylation at specific CpG sites contributes to the onset, severity, and progression of psychiatric disorders in own target individuals. These findings extend across a range of psychiatric disorders, including SCZ, BD, anxiety disorders, and other psychiatric disorders. Furthermore, MRSs provide a way of exploring tissue-specific methylation patterns that are critical for understanding the distinct epigenetic changes that may occur in the brain versus peripheral tissues.

## Procedures for the MRS Calculation

The MRS calculation involves several steps, each of which ensures the accurate identification and quantification of methylation patterns associated with psychiatric disorders. Below is an outline of these steps:

1. Discovery EWAS to Identify Differentially Methylated Positions

The first step in calculating MRSs involves conducting a discovery EWAS to identify the differentially methylated positions (DMPs) associated with psychiatric disorders using raw intensity data (IDAT) files or by obtaining the summary statistics of the EWAS from previous studies. The discovery EWAS examines the methylation patterns in various tissues, such as blood or postmortem brain tissues (e.g., prefrontal cortex, superior temporal gyrus, and hippocampus). DMPs represent the regions of the genome where methylation levels differ between patients with psychiatric disorder and healthy controls, providing clues about how gene expression may be altered in psychiatric disorders. By comparing the methylation profiles between groups, EWAS can identify the CpG sites strongly associated with the disorder risk, severity, or environmental exposures, such as childhood adversity and lifestyles.

2. Selection of Risk Methylation Sites in Discovery EWAS

To calculate MRSs, methylation CpG sites marginally associated with psychiatric disorders are selected based on various significance thresholds (*P_T_*), such as *P_T_* < 0.001, *P_T_* < 0.01,* P_T_* < 0.05, *P_T_* < 0.1, *P_T_* < 0.5, and *P_T_* ≤ 1, from the discovery EWAS. This selection process ensures that the MRS captures both strongly and moderately associated methylation changes, providing a more comprehensive assessment of the disorder’s epigenetic patterns. By varying *P_T_* thresholds, a broader range of methylation sites can be included in the MRS calculation, capturing subtle, but meaningful epigenetic contributions to the psychiatric disorder risk.

3. Methylation Data Processing in Target Cohort

After identifying the DMPs related to psychiatric disorders in the discovery EWAS cohort, the next step is to process the methylation data in the target cohort. DNA methylation is quantified using platforms, such as Illumina’s Human Methylation 450 or EPIC arrays, which measure the methylation intensity at specific CpG sites. Raw IDAT from methylation probes are normalized to reduce the technical variation, ensuring accurate comparisons across samples using the R package, such as ‘*meffil’* (efficient algorithms for analyzing the DNA methylation data). Poor-quality samples are excluded based on predefined criteria, such as bead number or detection *p*-values, to maintain data integrity. Furthermore, if the focus is on methylation patterns in whole blood, the six blood cell types, B cells, CD4+ T cells, CD8+ T cells, monocytes, neutrophils, and natural killer cells, can be estimated from the methylation data using *meffil’*s reference profile ^(35)^. In contrast, if the focus is on the postmortem brain tissue, the two cell types, neuronal (NeuN+) and nonneuronal (NeuN−) cells, can be estimated using *meffil’*s “guintivano dlpfc” reference profile. These cell counts can then be used as covariates to control for cell-type composition effects.

4. CoMeBack Method for the Methylation Data

To avoid redundancy in the methylation data within the target cohort, the CoMeBack method is applied ^(36)^, similar to a pruning procedure in the PRS calculation. This method identifies comethylated regions (e.g., based on a Spearman correlation cutoff of 0.3 and the proximity of CpG sites within a 2 kb proximity window) ^(33)^ and removes the correlated methylation sites, ensuring that only independent and non-overlapping methylation changes are included in the MRS calculation. This step is crucial for avoiding an overrepresentation of particular genomic regions and providing a more comprehensive representation of the methylation patterns.

5. MRS Calculation in the Target Cohort

Finally, MRSs in the target cohort are calculated by summing the methylation levels of the selected CpG sites, with each site weighted according to its effect size (*β* value) from the discovery EWAS. This weighted sum reflects the cumulative contribution of the methylation changes to the disorder, allowing for a quantification of an individual’s epigenetic risk for developing a psychiatric disorder. For instance, MRSs for psychiatric disorder can be compared between the target cohort of patients and controls. By integrating multiple methylation sites, MRSs provide a more comprehensive understanding of how epigenetic factors contribute to the psychiatric disorder risk.

## Limitations of the MRS Calculation and Availability of IDAT Files in Psychiatric Research

One of the primary limitations of the MRS calculation is the lack of large-scale EWAS datasets, similar to the GWAS resources available for the PRS calculation. Unlike GWAS, where large consortia, such as the PGC and UKBB, provide publicly accessible summary statistics, the EWAS summary statistics are not as widely available. Although repositories, such as the Gene Expression Omnibus (GEO), provide processed methylation data (e.g., txt or csv files), these datasets often vary in their preprocessing methods, resulting to challenging secondary use and meta-analyses. In contrast, raw IDAT files may be available for parts of studies through the GEO, but we must often conduct their own EWAS to generate the summary statistics. This variability and the lack of standardized, publicly available summary statistics limit the widespread application of MRSs and complicate efforts to replicate and validate findings across different studies.

To address this limitation, we searched the GEO for psychiatric epigenetic studies that have made IDAT files publicly available. Using specific search terms, such as (“schizophrenia” OR “schizoaffective” OR “delusional”) AND (“methylation”) for schizophrenia (275 hits), (“bipolar” OR “bipolar disorder”) AND (“methylation”) for bipolar disorder (458 hits), (“depression” OR “major depressive disorder” OR “MDD”) AND (“methylation”) for depressive disorder (549 hits), (“autism” OR “autism spectrum disorder” OR “ASD”) AND (“methylation”) for autism spectrum disorder (77 hits), (“attention deficit hyperactivity disorder” OR “ADHD”) AND (“methylation”) for attention deficit hyperactivity disorder (three hits), (“panic” OR “panic disorder” OR “agoraphobia”) AND (“methylation”) for panic disorder (seven hits), (“social anxiety disorder” OR “social phobia”) AND (“methylation”) social anxiety disorder (one hit), (“generalized anxiety”) AND (“methylation”) for generalized anxiety (zero hit), (“phobia” OR “phobias”) AND (“methylation”) for specific phobia (one hit), (“anxiety disorder”) AND (“methylation”) for anxiety disorders (392 hits), (“obsessive compulsive disorder” OR “OCD”) AND (“methylation”) for obsessive compulsive disorder (one hit), and (“post-traumatic stress disorder” OR “PTSD”) AND (“methylation”) for post-traumatic stress disorder (nine hits) until September 22, 2024, we compiled a list of studies providing IDAT files and summarized the findings, including the sample sizes and array types, in Table 1. This table provides a valuable resource for researchers aiming to perform their own EWAS and MRS calculations, facilitating greater accessibility to raw data for epigenetic research in psychiatric disorders. However, note that the table includes studies utilizing different array types, such as 27K, 450K and EPIC, as well as those with small sample sizes, some with fewer than ten participants.

**Table 1.** Raw Methylation Intensity Data (IDAT) Files Related to Psychiatric Disorders.

PMID	Series accession	Diagnosis	Case *n*	Control *n*	Tissue	Methylation array
39333502	GSE237561	SCZ (treatment-resistant)	26	0	Peripheral blood	450K or EPIC
35027166	GSE191200	SCZ, BD, MDD	1 SCZ, 3 BD, 10 MDD	8	Microglia from four brain regions^a^	EPIC
35879030	GSE157252	SCZ spectrum disorders^b^	84	50	Peripheral blood	EPIC
35987687	GSE144910	SCZ	44	44	Superior temporal gyrus	EPIC
31053723	GSE112179	SCZ, BD	29 SCZ, 27 BD	28	Frontal cortex	EPIC
30468562	GSE120342	SCZ, BD	3 SCZ, 4 BD	4	Dorsolateral prefrontal cortex	27K
26619358	GSE74193	SCZ	135	365^c^	Prefrontal cortex	450K
24399042	GSE61107	SCZ	24	24	Frontal cortex	450K
31746071	GSE129428	BD	32 BD-I	32	Hippocampus	EPIC
35027166	GSE191200	SCZ, BD, MDD	1 SCZ, 3 BD, 10 MDD	8	Microglia from four brain regions^a^	EPIC
31053723	GSE112179	BD, SCZ	27 BD, 29 SCZ	28	Frontal cortex	EPIC
30468562	GSE120342	BD, SCZ	4 BD, 3 SCZ	4	Dorsolateral prefrontal cortex	27K
26913521	GSE77135	BD	2	4	Scalp and dura mater tissues	450K
35858435	GSE201287	MDD^d^	40	40	Peripheral blood	450K
35027166	GSE191200	SCZ, BD, MDD	1 SCZ, 3 BD, 10 MDD	8	Microglia from four brain regions^a^	EPIC
28556790	GSE98203	Suicide	22	28	Orbitofrontal cortex	450K
34573415	GSE164563	ASD^e^	66	0	Peripheral blood	450K
31681403	GSE131706	ASD	17	17	Subventricular zone of the lateral ventricles	450K
31541176	GSE109905	ASD	38	31	Peripheral blood	450K
31379474	GSE108785	ASD monozygotic discordant twins	3	3	Peripheral blood	450K
32008165	GSE80017	ASD	9	9	Prefrontal cortex	450K
27404287	GSE83424	ASD	53	10	Peripheral Blood	450K
35145374	GSE186339	ADHD monozygotic discordant twins	2	2	Saliva	EPIC
35477560	GSE201016	PD^f^	183	85	Peripheral blood	450K
29249830	GSE102468	PD^g^	89	76	Peripheral blood	450K
33542190	GSE164056	SAD	66	77	Peripheral blood	EPIC
34717534	GSE148021	OCD	8	8	Cortical and ventral striatum areas^h^	450K
34239411	GSE172464	PTSD monozygotic discordant twins	6	6	Peripheral blood	EPIC
28696412	GSE89218	PTSD	48	51	Peripheral blood	450K

NA, not available; SCZ, schizophrenia; BD, bipolar disorder; MDD, major depressive disorder; ASD, autism spectrum disorder; ADHD, attention deficit hyperactivity disorder; PD, panic disorder; SAD, social anxiety disorder; OCD, obsessive compulsive disorder; and PTSD, post-traumatic stress disorder. ^a^Four brain regions, namely, medial frontal gyrus, superior temporal gyrus, subventricular zone, and thalamus. ^b^SCZ spectrum disorders, namely, SCZ, schizophreniform disorder, psychotic disorder not otherwise specified, brief psychotic disorder, or delusional disorder. ^c^Note that control groups included fetal, infant, and pediatric samples. ^d^Methylation-based sex differed from the demographic data sex. Additionally, note that the array plates were completely different between cases and controls. ^e^ASD severity was assessed using the Childhood Autism Rating Scale (CARS), and internalizing and externalizing symptoms were evaluated using the Child Behavior Checklist (CBCL). ^f^MDD replication samples are not publicly available. ^g^The PD sample comprises the same cohorts used in PMID 35477560. ^h^Cortical (anterior cingulate gyrus and orbitofrontal cortex) and ventral striatum (nucleus accumbens, caudate nucleus and putamen) areas.

## MRS for Schizophrenia and Bipolar Disorder

Genetic and environmental factors contribute to the pathogenesis of both SCZ and BD ^(37)^. Individuals in high-risk genetic groups, as determined by PRS deciles for SCZ, BD, and disorder-specific risks, exhibited more severe cognitive impairments, particularly in SCZ ^(20)^. To further explore the complex genetic and epigenetic mechanisms underlying SCZ and BD, we calculated MRSs for the SCZ risk based on EWASs derived from both blood samples and postmortem brain tissues, such as the frontal cortex and the superior temporal gyrus. We then investigated whether MRSs for the SCZ risk derived from the blood and brain tissues could further explain the genetic risk for SCZ, particularly in 66 patients with SCZ and 30 healthy controls stratified by PRS deciles for SCZ, BD, and disorder-specific risks ^(33)^.

The genetic SCZ risk group stratified by PRSs displayed more genome-wide significant DMPs compared with the genetic BD risk group, indicating that DMPs are particularly useful in distinguishing SCZ from BD at an epigenetic level, even after PRS stratification. Moreover, MRSs for the SCZ risk derived from the whole blood, frontal cortex, and superior temporal gyrus were higher in SCZ patients, particularly in those with a high genetic risk for SCZ compared to healthy controls. MRSs in the high-risk group were significantly elevated compared to both healthy controls and SCZ patients with a genetic risk for BD. Interestingly, no correlations were found between MRSs and PRSs, indicating that MRSs capture independent aspects of the SCZ risk that are not explained by genetic variation alone. These findings suggest that MRSs derived from both the blood and brain tissues can provide valuable insights into the epigenetic characteristics for SCZ, especially when the genetic risk is considered.

## MRS for Social Anxiety Disorder and Panic Disorder

Social anxiety disorder (SAD) and panic disorder (PD) are common anxiety disorders influenced by a complex relationship of genetic and environmental factors. These anxiety disorders share overlapping features and frequently co-occur, but also display distinct clinical characteristics. Both environmental factors and genetic predispositions contribute to their development. We recently calculated MRSs to assess their association with the SAD and PD risks and the severity of social anxiety symptoms, as well as with the environmental factors, such as childhood adversity and overall stressful life events ^(32)^. Preliminary EWASs for the SAD risk, severity of social anxiety symptoms, childhood adversity in SAD, PD risk, and impact of overall stressful life events in PD were conducted to identify DMPs in individuals with SAD and PD compared to healthy controls (HCs), that is, 66 individuals with SAD and 77 HCs ^(14)^ and 182 PD individuals and 81 HCs ^(15)^, using publicly available raw IDAT files.

MRSs derived from the EWAS for the SAD risk and the severity of social anxiety symptoms were significantly higher in PD patients compared to HCs ^(32)^, indicating that some epigenetic changes associated with SAD may also play a role in PD pathogenesis. Interestingly, MRSs related to overall stressful life events, particularly in PD individuals, were lower in patients with SAD compared to HCs ^(32)^, suggesting that the influence of the overall stressful life events on methylation patterns may differ between SAD and PD. These findings highlight both the shared and distinct epigenetic features of SAD and PD. The shared methylation patterns suggest that common pathways may underlie aspects of both anxiety disorders. At the same time, disorder-specific epigenetic signatures, such as those related to social avoidance in SAD, provide important perspectives on the unique mechanisms contributing to each disorder. By integrating MRSs, we can understand better the epigenetic characteristics of anxiety disorders, which may help in identifying the novel biomarkers and therapeutic targets for SAD and PD. However, these findings must be interpreted with caution, given the limited scale of current epigenetic studies on SAD and PD.

## Applications of MRS in Psychiatric Research

The application of MRSs in psychiatric research has opened new avenues for understanding the complex relationship between genetic and environmental factors in psychiatric disorders. MRSs provide a quantitative framework for assessing cumulative epigenetic modifications, offering perspectives on how these changes influence the onset, severity, and progression of psychiatric disorders. By integrating data from EWASs, MRSs help identify common and distinct methylation patterns associated with genetic risk and environmental exposures, such as early-life adversity and chronic stress.

One of the promising, yet complex applications of MRSs is in early detection and risk stratification. While MRSs can help identify individuals with increased risk, their predictive utility for distinguishing psychiatric conditions from healthy states remains modest. MRSs may help indicate a higher risk for psychiatric disorders, potentially enabling early interventions that could mitigate the onset or progression of these disorders. In addition, MRSs show potential as biomarkers for psychiatric disorders, providing a valuable understanding of both the biological underpinnings of these disorders and the influence of environmental factors on gene regulation. Nonetheless, ethical considerations are crucial in applying MRSs for early intervention, particularly regarding the prediction accuracy and their implications. As more data from large-scale EWASs become available, MRSs may eventually be utilized in personalized medicine, guiding treatment decisions based on an individual’s unique methylation profile and providing a more tailored therapeutic approach.

## Conclusion

MRSs represent a powerful tool in psychiatric research, offering a complementary perspective to PRSs by focusing on the epigenetic modifications influenced by environmental factors. While PRSs capture the static genetic risk, MRSs provide both static and dynamic understanding of how environmental factors may modulate gene expression over time. By integrating data from EWASs and applying MRS calculations, we can understand better the complex genetic and epigenetic mechanisms underlying psychiatric disorders. Despite the MRS promise, their application in clinical settings remains challenging because of the lack of large-scale EWAS datasets and standardized summary statistics that complicates replication and validation across different studies. Nevertheless, as more MRS studies are being conducted, MRSs are likely to become an integral part of psychiatric research and may offer additional perspectives on clinical practice.

## Article Information

This article is based on the study, which received the Medical Research Encouragement Prize of The Japan Medical Association in 2024.

### Conflicts of Interest

None

### Sources of Funding

This work was supported by the Grants-in-Aid for Scientific Research (C) (21K07497 and 22K07614) from the Japan Society for the Promotion of Science (JSPS), the AMED under grant numbers JP21uk1024002, JP22dk0307112, and JP23dk0307103, a grant from the Takeda Science Foundation (N/A), and a grant from the Mochida Memorial Foundation for Medical and Pharmaceutical Research (N/A).

### Author Contributions

KO supervised the entire project, collected the data, wrote the manuscript, and was critically involved in the design and data interpretation. KO, DF, KT, AK, and YM performed the literature review. KO, DF, SS, and TS were heavily involved in the collection of the majority of the data and intellectually contributed to the data interpretation. All authors contributed to and approved the final manuscript.

### Approval by Institutional Review Board (IRB)

Not applicable.
